# Exploration of Active Compounds and Potential Mechanisms of Ginseng Dripping Pills: An Integrated Study Utilizing UHPLC‐Q‐Orbitrap MS Analysis and Network Pharmacology

**DOI:** 10.1155/ianc/6693812

**Published:** 2026-09-28

**Authors:** Minglin Xu, Junyi Mao, Xiangnan Wang, Jinping Jiang, Simon Sani Ocholi, Chaoyang Wang, Yue Wang, Honghai Yu, Lifeng Han

**Affiliations:** ^1^ State Key Laboratory of Component-Based Chinese Medicine, Tianjin Key Laboratory of TCM Chemistry and Analysis, Haihe Laboratory of Modern Chinese Medicine, Tianjin University of Traditional Chinese Medicine, Tianjin 301617, China, tjutcm.edu.cn; ^2^ Lishen Pharmaceutical Co., Ltd., Tonghua 134000, Jilin, China

**Keywords:** ginseng dripping pills, GNPS, network pharmacology, UHPLC-Q-Orbitrap MS

## Abstract

Ginseng dripping pills (GDPs), a traditional Chinese patent medicine, are extensively used for cardiovascular diseases (CVDs), but their active components and underlying mechanisms remain unclear. This study aimed to identify the chemical constituents of GDP and to explore their potential mechanisms in treating myocardial infarction (MI) and atherosclerosis (AS). Chemical profiling was performed using ultra‐high‐performance liquid chromatography coupled with Q‐Exactive^TM^ hybrid quadrupole–Orbitrap mass spectrometry (UHPLC‐Q‐Orbitrap MS) and the Global Natural Products Social Molecular Networking (GNPS), leading to the identification of 113 compounds, and 13 blood‐absorbed components were identified, with ginsenosides identified as the predominant bioactive constituents. Network pharmacology was then used to predict the key therapeutic targets and associated signaling pathways, and a comprehensive “GDP–component–target–pathway” interaction network was constructed. 18 targets were identified as core targets of GDP in treating CVD. The main pathways involved included PI3K‐Akt, RAS, cAMP, Rap1, and calcium signaling. Molecular docking showed that 13 blood‐absorbed components of GDP, including ginsenoside Ro, ginsenoside Rs1 and ginsenoside Ra3, had strong binding affinities with MAPK3, MMP9, and PTGS2. These findings suggest that GDP may exert cardioprotective effects through a multicomponent, multitarget, and multipathway mode of action, providing a scientific basis for its clinical application in CVD treatment.

## 1. Introduction

As one of the world’s leading causes of death, cardiovascular disease (CVD) is linked to high rates of morbidity, mortality, disability, and various complications [1], thereby posing a significant burden on global public health. It is estimated that the annual number of deaths attributable to CVD will approach 24 million by 2030 [2], with heightened risk driven by population aging; individuals aged ≥ 65 years are expected to comprise approximately one‐quarter of the global population by 2035 [3]. The etiology of CVD is multifactorial, involving unhealthy lifestyles, environmental contamination, and genetic predispositions, and it frequently coexists with comorbidities such as diabetes, hypertension, hyperlipidemia, obesity, and psychosocial stress [4]. The spectrum of CVD includes coronary heart disease; hypertension; heart failure; arrhythmia; cardiomyopathy; congenital heart disease; as well as valvular, pericardial, and endocardial diseases. These diseases can severely impair cardiac function.

Myocardial infarction (MI), a severe form of acute coronary syndrome, is primarily caused by coronary artery disease, leading to an abrupt reduction in coronary blood flow and persistent myocardial ischemia [5–7]. Despite global declines in MI‐related mortality, heart failure mortality and complication rates remain considerable [8]. Atherosclerosis (AS), a chronic inflammatory process underpinning many CVDs, such as MI [9, 10], is characterized by lipid accumulation, inflammation, and plaque formation. With the rapid development of China’s population aging, AS morbidity and mortality are expected to increase. Since existing lipid‐lowering therapies fail to fundamentally prevent CVD, the development of effective therapeutic approaches for AS remains of great importance [11].

Traditional Chinese medicine (TCM) has demonstrated significant potential in the treatment of CVD, largely due to its multicomponent and multitarget properties [12]. One notable example is ginseng dripping pills (GDPs), a modern patent TCM formulation derived from *Panax ginseng*. These dripping pills are produced through a water–alcohol extraction method. After cooling and solidification, they are formed into small, oil‐coated spheroidal units designed for convenient oral administration. By integrating traditional formulations with modern pharmaceutical technology, GDP improves the bioavailability of key active constituents, particularly ginsenosides and triterpenoid saponins, widely recognized for their antioxidant, anti‐inflammatory, and anti‐CVD activities [13].

To further investigate the active ingredients of TCM and their mechanisms of action, the Global Natural Products Social Molecular Networking (GNPS) (https://gnps.ucsd.edu/) combined with mass spectrometry (MS), particularly liquid chromatography–MS (LC‐MS), has become an indispensable research tool. GNPS is a robust online database platform designed for the analysis of MS data from natural products and the construction of molecular networks. This platform computes modified cosine scores to evaluate pairwise spectral similarities within fragment spectra. The results are then visualized as a network graph, thereby facilitating the identification of compounds and their analogs. Through molecular network construction, both known compounds can be annotated, and potential relationships among unknown compounds can be inferred. Therefore, GNPS serves as a powerful tool for in‐depth investigation of TCM [14–17].

Network pharmacology is a comprehensive analytical approach that integrates multiple techniques such as systems biology, high‐throughput screening, network analysis, and network visualization. It enables systematic evaluation of drug–disease interactions and helps elucidate the complex interaction system of “drug–component–target–disease.” This technology is frequently applied in TCM research to predict the biological functions of compounds and their potential targets. Consequently, it has become an important tool for basic research on the efficacy of modern TCM [18–20]. Molecular docking evaluates the interaction between ligands (compounds) and receptors (macromolecular proteins) within a three‐dimensional structure by calculating physicochemical and other parameters. This technology is instrumental in drug screening and has been extensively utilized in the studies of active ingredients and target‐specific compound preparations in TCM [21].

LC‐MS enables accurate identification of individual chemical constituents in TCM [22]. In drug discovery and production, MS is extensively employed for structure analysis, impurity analysis, and metabolism studies. Owing to its high sensitivity, resolution, and selectivity, LC‐MS is a powerful tool for characterizing the chemical composition of TCM and identifying active ingredients in complex formulations [23–25]. Ultra‐high performance LC–quadrupole‐Orbitrap MS (UHPLC‐Q‐Orbitrap MS) is an advanced analytical technique that integrates the superior separation capability of UHPLC with the accurate mass measurement and structural elucidation capabilities of Q‐Orbitrap MS. This technology is characterized by high resolution, high sensitivity, high throughput, and multimode analysis and is widely used in TCM analysis, drug discovery, environmental pollutant analysis, and other fields [26–29].

The aim of this study was to systematically characterize the chemical constituents of GDP and to identify the bioavailable components that are absorbed into systemic circulation after oral administration, thereby clarifying the pharmacodynamic material basis and potential mechanisms underlying its therapeutic effects.

To achieve this, UHPLC‐Q‐Orbitrap MS combined with GNPS‐based molecular networking was employed for the rapid and comprehensive characterization of the chemical constituents in GDP. Subsequently, blood‐absorbed components were identified through plasma analysis, as only compounds that enter systemic circulation are considered to contribute directly to the in vivo pharmacological effects of GDP. Based on the compound identification and the network pharmacological correlation analysis, the “GDP–bioactive compound–target–disease” interaction network was successfully constructed. Furthermore, molecular docking was utilized to validate the rationality of the established network. The overall analytical workflow of this study is illustrated in Figure 1. This integrated strategy provides a systematic framework for investigating the pharmacodynamic material basis and mechanisms of GDP and offers a reference for its quality control and clinical application.

**FIGURE 1:**
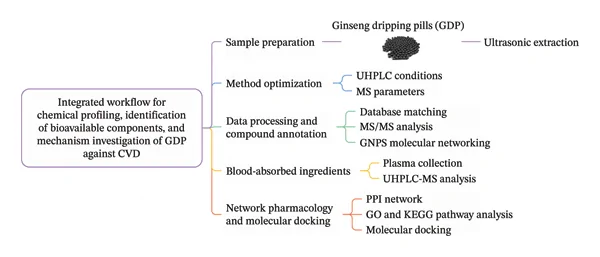
Schematic workflow of the integrated analytical and computational strategy used in this study.

## 2. Experimental Section

### 2.1. Chemicals and Reagents

The reference standards utilized in this study, including arginine, ginsenoside Rh4, ginsenoside Rg5, ginsenoside Rd, ginsenoside Rb1, pseudoginsenoside F11, ginsenoside Rg1, ginsenoside Re, and ginsenoside Rh2, were obtained from Chengdu Mansion Biotechnology Co., the China National Institute for the Control of Pharmaceutical and Biological Products, and Shanghai Yuanye Biotech Co. The purity of all standards was higher than 98%.

The experimental reagents consisted of HPLC‐grade acetonitrile (CH_3_CN), methanol (MeOH) (Fisher, Fair Lawn, NJ, USA), and formic acid (FA) (ACS, Wilmington, DE, USA), as well as acetic acid (HOAc) (Sigma‐Aldrich, St. Louis, MO, USA). Deionized water was obtained from Watson Distilled Water.

### 2.2. Standard Solutions and Sample Preparation

Individual stock standard solutions were prepared at 1 mg/mL, and then mixed and diluted to obtain a mixed standard solution at a concentration of 10 μg/mL. All prepared solutions were preserved at 4°C for further analysis.

GDP was supplied by Lishen Pharmaceutical Co., Ltd. (Tonghua, Jilin, China) and ground into a fine powder. The composition of GDP includes polyethylene glycol (PEG) 4000, PEG 6000, and ginseng total extract at the ratio of 4:6:5, respectively. Four samples of GDP powder (0.5 g each) were accurately weighed and separately dissolved in 10 mL of different solvents, including purified water, 30% methanol (*v/v*), 60% methanol (*v/v*), and methanol. Each sample was subjected to ultrasonic extraction for 120 min.

### 2.3. Animals and Treatment

Five male Sprague–Dawley rats (220 ± 10 g), certified as specific pathogen–free (SPF), were procured from Beijing HFK Bioscience Co., Ltd. (Beijing, China). The animals were housed at the Animal Center of Tianjin University of TCM (Tianjin, China) and acclimated for one week under controlled conditions (temperature: 24 ± 2°C; humidity: 40%–60%; 12‐h light/dark cycle) with free access to standard rodent diet and water. Prior to sampling, all rats were subjected to a 24‐h fasting period but had free access to water.

A self‐controlled study design was adopted, with each individual rat considered as the experimental unit, to reduce individual differences. Blood samples collected before drug administration were defined as the control (non‐GDP) group, and those collected after administration were defined as the GDP‐administered group. A GDP suspension (4 g/kg) was administered via oral gavage. At 5, 10, 30, 60, 120, and 240 min after administration, blood was collected from the retro‐orbital plexus into heparinized tubes. These time points were chosen to see the absorption of GDP. Samples from each rat were first processed separately. Subsequently, plasma samples from different time points were pooled into two groups (0‐1 h and 2–4 h) for further analysis. This allowed us to qualitatively compare the overall distribution pattern of blood‐absorbed components over time. After the final blood collection, rats were euthanized by intraperitoneal injection of pentobarbital sodium (200 mg/kg). After confirming the absence of vital signs, cervical dislocation was performed by trained personnel to ensure complete euthanasia.

All experimental protocols received approval from the Institutional Animal Care and Use Committee of Tianjin University of TCM (TCM‐LAEC2024051x1132) in compliance with AAALAC International standards.

### 2.4. UHPLC‐Q‐Orbitrap MS Analysis

The multicomponent profiling and characterization of GDP were performed on an UltiMate 3000 UHPLC (Thermo Fisher Scientific, San Jose, CA, USA). A Waters ACQUITY UPLC BEH C_18_ column (2.1 mm × 100 mm, 1.7 μm) was used for chromatographic separation at a column temperature of 35°C. The UHPLC system was interfaced with a Q Exactive^TM^ hybrid Q‐Orbitrap MS system (Thermo Fisher Scientific) to obtain high‐accuracy MS data. The mobile phase consisted of water containing 0.2% HOAc (A) and CH_3_CN (B), delivered at 0.3 mL/min. Each sample was injected at a volume of 3 μL. The gradient elution program was set as follows: 0‐1 min, 5%–10% B; 1–20 min, 10%–100% B; 20–22.5 min, 100% B; 22.5–23 min, 100%–5% B; and 23–25.5 min, 5% B. In addition, during method development, a shorter gradient program (11 min) was used for rapid screening of GDP samples; however, the optimized 25.5 min method was ultimately used for comprehensive component profiling.

High‐resolution MS spectra data were acquired in both positive and negative ionization modes using a heated electrospray ionization (HESI) source. The source parameters were as follows: spray voltage: −3.0/+3.0 kV, sheath gas: 35 arb, auxiliary gas: 10 arb, capillary temperature: 360°C, and auxiliary gas heater temperature: 450°C. The Orbitrap analyzer scanned over an *m*/*z* range of 100–1500. Normalized collision energy (NCE) was set as 10/20/40 V.

All total ion chromatograms (TICs) were processed with Thermo Fisher Xcalibur 4.0 software (Thermo Fisher Scientific) for peak identification, matching, alignment, and normalization. The mass error threshold was controlled within ± 5 ppm.

### 2.5. Plasma Sample Treatment

All blood samples were immediately centrifuged at 7000 × *g* for 10 min at 4°C using an Eppendorf 5424R centrifuge (Barkhausenweg, Hamburg, Germany). The supernatant was stored at −80°C for further analysis. For sample preparation, 200 μL plasma aliquots were mixed with fourfold volumes of cold methanol, vortexed for 5 min, and centrifuged at 13200 × *g* for 10 min at 4°C. The obtained supernatant was transferred to new tubes and evaporated to dryness under a gentle nitrogen stream. The residue was re‐dissolved in 50 μL of 50% methanol–water (*v/v*), vortexed for 5 min, and centrifuged at 13200 × *g* for 20 min at 4°C. Finally, 40 μL of the supernatant was transferred to HPLC vials, and 3 μL of the sample was injected for analysis.

### 2.6. GNPS Molecular Network Construction

The molecular network was constructed using the online workflow of the GNPS platform. Raw data acquired from UHPLC‐Q‐Orbitrap MS were first converted into mzML format with MSConvert, and then uploaded to the GNPS server via WinSCP 6.3.3. The network was constructed based on MS/MS spectral similarity. The parameter configuration is as follows: precursor ion mass tolerance = 0.02 Da, fragment ion mass tolerance = 0.02 Da, cosine score threshold = 0.7, and minimum matched fragment ions = 6, with all other parameters at default settings.

After data processing, the acquired spectra were automatically compared with the GNPS public spectral libraries (GNPS community knowledge base of reference spectra) for spectral matching. The resulting molecular network was exported and visualized using Cytoscape 3.9.1, where nodes represent precursor ions and edges indicate spectral similarity. Based on the clustering results, compounds within the same molecular family were further analyzed by integrating database searches and literature reports to support structural annotation.

### 2.7. Network Pharmacology Analysis

#### 2.7.1. Prediction of Therapeutic Targets of GDP Against CVD

According to the principle of serum pharmacochemistry, only constituents absorbed into the systemic circulation after oral administration can potentially reach target organs and exert pharmacological effects [30–32]. Therefore, prototype compounds identified in rat plasma following oral gavage of GDP were considered as candidate bioactive components. Their SMILES identifiers obtained from the PubChem database (https://pubchem.ncbi.nlm.nih.gov/) were submitted to SwissTargetPrediction (https://www.swisstargetprediction.ch/) to forecast potential therapeutic targets. Disease‐related targets for MI and AS were retrieved from the Online Mendelian Inheritance in Man (OMIM) database (https://omim.org/), GeneCards database (https://www.genecards.org/), Therapeutic Target Database (TTD) (https://db.idrblab.net/ttd/), PharmGKB database (https://www.pharmgkb.org/), and DrugBank database (https://go.drugbank.com/); duplicates in both drug and disease targets were removed prior to intersection analysis. Venn analysis identified shared targets between GDP compounds and CVD. These overlapping target genes were imported into the STRING database (https://cn.stringdb.org/), with the species restricted to “Homo sapiens” to ensure physiological relevance to human CVD. A high‐confidence interaction score (> 0.7) was used to construct a protein–protein interaction (PPI) network, thereby improving specificity and reducing potential false‐positive interactions. The resulting PPI network was visualized using Cytoscape 3.9.1.

#### 2.7.2. Gene Ontology (GO) and Kyoto Encyclopedia of Genes and Genomes (KEGG) Pathway Analyses

Functional enrichment analysis was performed using the Database for Annotation, Visualization, and Integrated Discovery (DAVID) (https://davidbioinformatics.nih.gov/). This included GO analysis spanning molecular functions (MFs), biological processes (BPs), and cellular components (CCs), alongside KEGG pathway analysis. Significantly enriched terms (*p* < 0.05) were clustered and ranked by *p* value. The top 10 GO terms and top 20 KEGG pathways were subsequently visualized to illustrate key biological patterns.

#### 2.7.3. Network Construction of the “GDP–Bioactive Ingredient–Target–Pathway–Disease” and Molecular Docking of Key Bioactive Compounds and Targets

A comprehensive network linking GDP bioactive compounds, targets, pathways, and diseases was constructed using Cytoscape 3.9.1 by integrating bioactive components of GDP, their associated disease targets, and the top 15 KEGG pathways.

Molecular docking was performed to investigate interactions between bioactive compounds and target proteins. The chemical information of key bioactive compounds was obtained from PubChem, and their three‐dimensional structures were generated in ChemDraw3D 23.1.1, and the lowest‐energy conformations were converted into PDBQT format using OpenBabel3.1.1. Core target proteins were obtained from the Protein Data Bank (PDB) (https://www.rcsb.org/) and UniProt. Protein structures were preprocessed in PyMOL 2.3.0 by removing solvent molecules and organic ligands. Subsequently, polar hydrogens were added, Gasteiger charges were computed, and AD4 atom types were assigned in AutoDockTools, followed by conversion to PDBQT format.

Docking simulations were carried out with AutoDock Vina 1.2.0. A blind docking strategy was employed, in which the grid box was defined to cover the entire protein, so as to achieve unbiased exploration of potential binding sites. Then, docking calculations were performed to obtain binding affinity values (kcal/mol) for the ligand–protein complexes. Grid box parameters were individually adjusted for each target to account for variations in protein size. Binding energies were visualized as heatmaps using GraphPad Prism 10.1.2. The top two docking conformations with the lowest binding energies were further visualized in PyMOL 2.3.0.

To further evaluate the reliability of the docking results, a re‐docking procedure was performed with AutoDock Vina 1.2.0. The co‐crystallized ligand was extracted from the protein structure and re‐docked into its original binding site using the same parameters. The top three ligand–protein complexes with the lowest binding energies were selected and re‐docked again under identical parameter settings. The root‐mean‐square deviation (RMSD) values between the original and re‐docked poses were calculated. An RMSD value below 2.0 Å was considered to indicate good agreement, suggesting stable and reliable predicted binding modes.

### 2.8. Statistical Analysis

The rat serum study was conducted using a self‐controlled design with five independent biological replicates (*n* = 5). Serum samples were collected before dosing as controls and at 5, 10, 30, 60, 120, and 240 min postadministration. Postadministration samples were pooled into two time intervals (0‐1 h and 2–4 h) for qualitative UHPLC‐Q‐Orbitrap MS profiling. As the analysis focused on compound identification, no statistical comparisons were performed.

For network pharmacology analysis, overlapping targets were identified using Venn analysis. PPI networks were constructed using the STRING database (interaction score > 0.7) and visualized in Cytoscape 3.9.1. Topological parameters, including degree, betweenness centrality, and closeness centrality, were calculated to identify key targets. Functional enrichment analyses (GO and KEGG pathways) were performed using the DAVID database. Statistical significance was evaluated using Fisher’s exact test, and terms with *p* < 0.05 were considered significant.

Molecular docking was carried out using AutoDock Vina 1.2.0, and binding energy values (kcal/mol) were used for comparative evaluation. Interactions with binding energies lower than −6.0 kcal/mol were considered indicative of favorable binding. Docking results were visualized as heatmaps using GraphPad Prism 10.1.2. To validate the reliability of the docking protocol, redocking was performed by re‐docking the co‐crystallized ligand into the active site of the target protein. The RMSD between the predicted and crystallographic conformations was calculated, and an RMSD value < 2.0 Å was considered acceptable.

## 3. Results

### 3.1. Optimization of Extraction Solvent

Given the complex composition of GDP, extraction efficiency plays a critical role in the characterization of its components. To identify the optimal extraction solvent, four different solvent systems: pure water, 30% methanol–water (*v/v*), 60% methanol–water (*v/v*), and methanol, were compared. As shown in Figure 2(a), the TICs revealed distinct extraction profiles for each solvent. Pure water preferentially extracted highly polar constituents, yielding strong elution peaks at early retention times (*t*_*R*_). In contrast, methanol‐containing solvents increasingly favored the extraction of less polar compounds, resulting in more pronounced peaks at mid to late *t*_*R*_. Notably, 60% methanol–water (*v/v*) provided a balanced extraction, capturing a wide range of polar and nonpolar compounds with well‐resolved chromatographic peaks.

**FIGURE 2:**
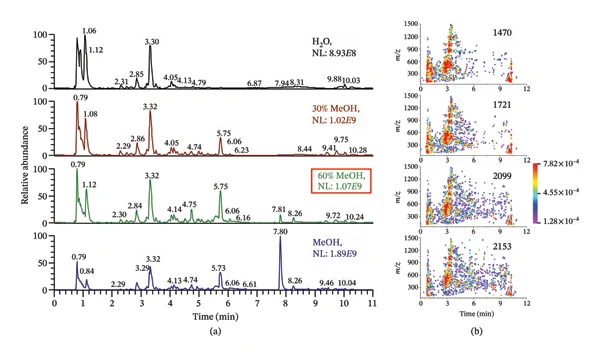
Optimization of extraction solvents. (a) TICs of solvents’ optimization conditions. (b) Number of extracted peaks.

As shown in Figure 2(b), we evaluated extraction performance through both chromatographic peak resolution in TICs and SIEVE‐analyzed component profiles. Scatter plots of peak distributions were color‐coded by relative intensity, with red representing higher compound abundance. Both 60% methanol–water (*v/v*) and 100% methanol extracted a greater number of compounds and exhibited broader *t*_*R*_ coverage compared to pure water and 30% methanol–water (*v/v*). Based on comprehensive assessment of extraction yield and separation efficiency, 60% methanol–water (*v/v*) was selected as the optimal solvent, delivering maximal compound coverage with well‐resolved chromatographic peaks, enhancing the reliability of subsequent qualitative and quantitative analyses.

### 3.2. Parameter Optimization of the UHPLC‐Q‐Orbitrap MS System

#### 3.2.1. Optimization of Chromatographic Parameters

A systematic optimization was performed on various chromatographic parameters, including stationary phase composition, mobile phase, flow rate, column temperature, and gradient elution scheme. The separation performance of five different columns (BEH C_18_, RP_18_, HSS T_3_, CSH C_18_, and Cortex C_18_) was comparatively evaluated. As shown in Figure S1, BEH C_18_ and HSS T_3_ columns demonstrated superior peak symmetry. Considering both the number of peaks extracted by SIEVE software and favorable retention characteristics with excellent resolution, the BEH C_18_ column was chosen for subsequent analyses.

As shown in Figure S2, adding a small amount of organic acid to the aqueous phase significantly improved peak shape, reduced tailing, and enhanced ionization efficiency compared to pure water. Further optimization (Figure S3) revealed that increasing HOAc concentration in the CH_3_CN–HOAc mobile phase resulted in a higher peak count and improved peak symmetry. Consequently, CH_3_CN–water containing 0.2% HOAc was adopted as the mobile phase. Column temperatures (30°C, 35°C, 40°C, and 45°C) were evaluated for separation efficacy (Figure S4). Optimal peak separation, symmetry, and peak capacity were achieved at 35°C. Flow rate analysis (Figure S5) showed that 0.3 mL/min provided superior peak morphology compared to 0.1 and 0.2 mL/min.

Final method optimization included gradient elution adjustments and sample concentration optimization to enhance chromatographic resolution and analyte response. The established separation protocol was achieved on a BEH C_18_ column using a CH_3_CN–0.2% HOAc mobile phase at 35°C with a flow rate of 0.3 mL/min (Figure S6).

#### 3.2.2. Optimization of MS Conditions

Preliminary characterization of GDP indicated that more components were detected in the negative ion mode. To maximize sensitivity and ensure reliable compound identification, we optimized collision energies and HESI source parameters for comprehensive acquisition of fragmentation spectra in negative ionization mode. The optimized UHPLC‐Q‐Orbitrap MS conditions were consistently applied to both GDP and plasma samples, with data acquired in both ionization modes for comprehensive analysis.

##### 3.2.2.1. Optimization of Collision Energy

To acquire comprehensive MS^2^ spectral data, NCE was systematically optimized at multiple settings, including 10/20/30 V, 10/20/40 V, 10/30/50 V, and 20/40/60 V. This range was selected to accommodate the diverse fragmentation requirements of compounds with varying abundances. Representative GDP constituents were selected as reference compounds for fragmentation optimization, including the following: protopanaxadiol‐type (PPD‐type) compounds (ginsenoside Rb1 and Rc), protopanaxatriol‐type (PPT‐type) compounds (malonyl floralginsenoside Re1 and Rg1), oleanolic acid–type (OA‐type) compounds (ginsenoside Ro), and maltol. As demonstrated in Figure 3(a), all compound classes generated characteristic fragments across the tested collision energies. The 10/20/40 V condition was ultimately chosen because it provided the highest fragment ion yield for most compounds while preserving structural information.

FIGURE 3Optimization of the Orbitrap MS in the negative ion mode. (a) Normalized collision energy. (b) Ion source parameters.

**Figure:**
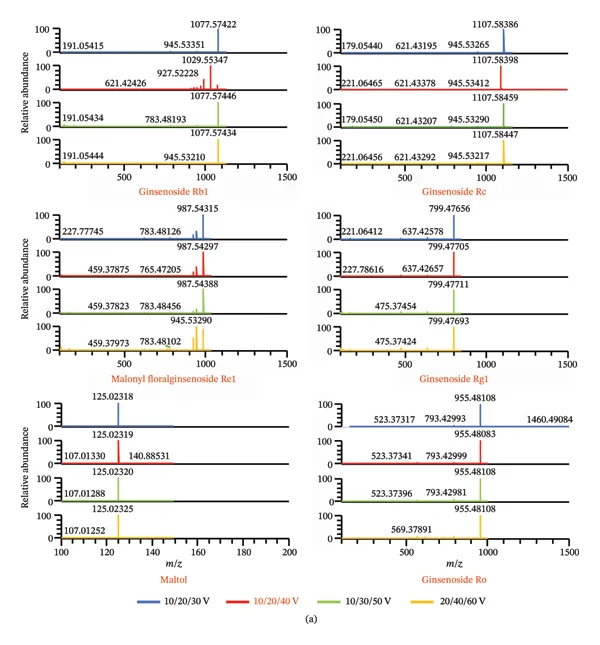

**Figure:**
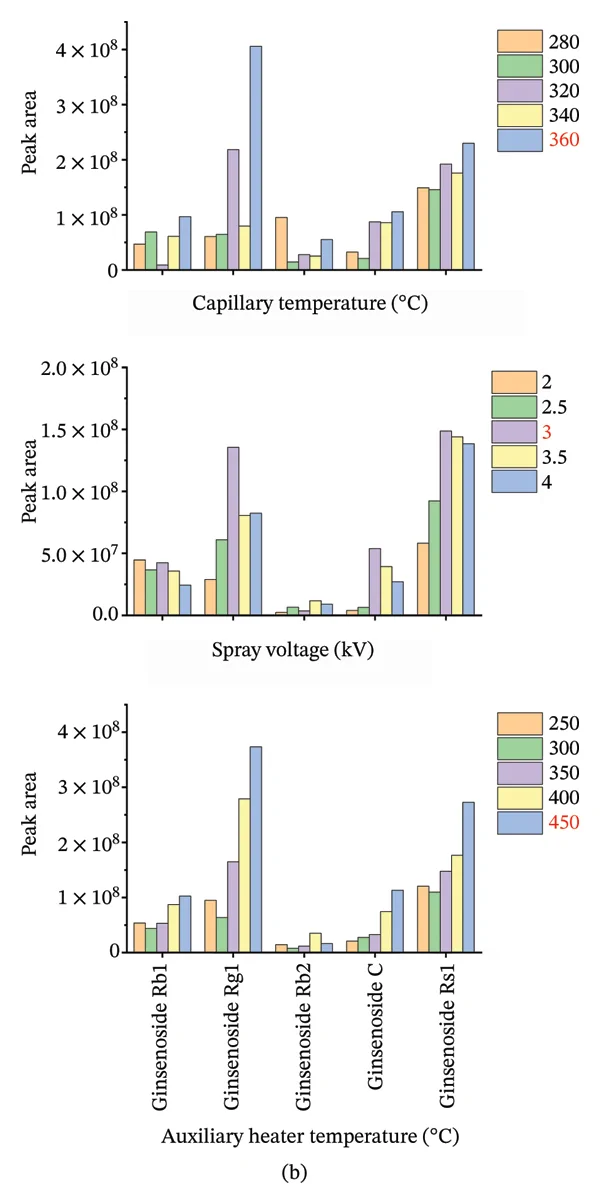

##### 3.2.2.2. Optimization of Ion Source Parameters

Representative ginsenosides (Rb1, Rg1, Rb2, Rc, and Rs1) from GDP were selected to optimize key ion source parameters, including spray voltage, capillary temperature, and auxiliary gas heater temperature, with peak area used as the evaluation criterion. In negative ion mode, capillary temperature was evaluated over the range of 280°C–360°C. Most compounds exhibited maximal peak areas at 360°C. For Rb2, the peak area at 360°C was slightly lower than that at 280°C but remained substantially higher than those observed at other temperatures (Figure 3(b)). Spray voltage optimization (2.0–4.0 kV in 0.5 kV increments) and auxiliary gas heater temperature (250°C–450°C, in 50°C increments) were further optimized. Except for Rb2, all analytes showed better responses at 3.0 kV and 450°C, respectively. The spray voltage of 3.0 kV was selected to balance sensitivity with discharge stability.

All optimizations are illustrated in Figure 3(b).

### 3.3. Molecular Network Analysis

The GDP molecular network was constructed and visualized in Figures S7–S10. It comprised 961 nodes and 26 clusters, with each node representing a unique molecule. Arrows between nodes reflect interaction strength and directionality. Molecular network annotation identified Comp. **37** as ginsenoside Rg1 through matches in quasimolecular ions, fragment ions, and relative retention time versus reference standards. The ginsenoside Rg1‐containing cluster (Figure 4) consisted of 62 nodes, exhibiting characteristic saponin fragments: PPD‐type (*m*/*z* 459.38 and 375.29), PPT‐type (*m*/*z* 475.38 and 391.29), and OA‐type (*m*/*z* 455.35) [28], confirming its classification as a ginsenoside analog. By matching experimental raw data with the database, 28 of these nodes were identified. The 4 nodes (Comp. **36**, ginsenoside Re; Comp. **37**, ginsenoside Rg1; Comp. **51**, pseudoginsenoside F11; and Comp. **100**, ginsenoside Rg5) were compared with reference standards for quasimolecular ion peaks, fragment ions, and relative *t*_*R*_, yielding consistent results. These findings facilitated the identification of the ginsenoside components in GDP.

**FIGURE 4:**
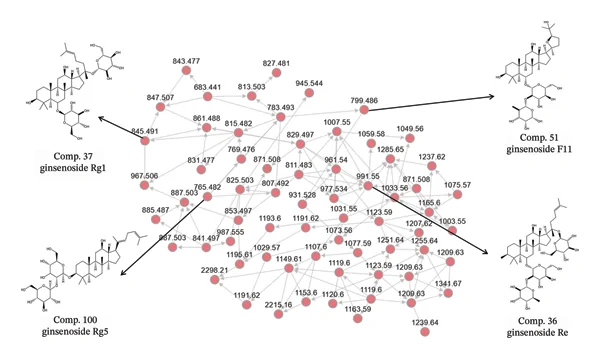
Ginsenosides identified from GDP using the GNPS database.

### 3.4. Composition Analysis of GDP

All raw data acquired under optimized analytical conditions were processed using Compound Discoverer 2.3.1 for peak characterization. A total of 113 compounds were identified through integrated data mining: 9 compounds via direct comparison of *t*_*R*_, MS, and MS/MS fragments with reference standards; 62 compounds through the Human Metabolome Database (HMDB) (https://hmdb.ca/) and PubChem database queries; and 43 compounds via GNPS platform matching. Identification criteria consistently incorporated quasimolecular ion masses, secondary fragmentation patterns, and chromatographic retention alignment. The remaining 11 compounds were designated as uncharacterized constituents requiring further structural validation. Comprehensive compound data are presented in Table 1.

**TABLE 1:** Identification of compounds in GDP obtained by Orbitrap MS.

Comp.	*t*_*R*_ (min)	*m*/*z*	Adduct ions	Formula	Mass error (ppm)	MS/MS (*m*/*z*)	Identification
1	0.73	175.1179	[M + H]^+^	C_6_H_14_N_4_O_2_	−0.01	130.0975 84.0808 70.0651 60.0556	Arginine^∗^

2	0.79	341.1089	[M − H]^−^	C_12_H_22_O_11_	3.11	161.0458 179.0564 119.0352 89.0233 71.0132 59.0128	Trehalose^#^

3	0.87	128.0341	[M − H]^−^	C_5_H_7_NO_3_	−0.93	128.0341 84.0444	4‐Oxoproline^#^

4	0.89	191.0180	[M − H]^−^	C_6_H_8_O_7_	−3.29	191.01800	Citric acid^#^

5	0.89	129.0182	[M − H]^−^	C_5_H_6_O_4_	−0.27	129.0182 85.0284 57.0335	Citraconic acid^#^

6	0.92	173.0076	[M − H]^−^	C_6_H_6_O_6_	−2.68	154.9975 129.0196 111.0077	Aconitic acid^#^

7	0.93	154.0982	[M + H]^+^	C_7_H_11_N_3_O	4.49	154.0982 112.0859	N‐Acetylhistamine isomer^†^

8	1.11	154.0980	[M + H]^+^	C_7_H_11_N_3_O	3.32	154.0980 112.0859	N‐Acetylhistamine^#^

9	1.12	128.0341	[M − H]^−^	C_5_H_7_NO_3_	−0.93	128.0341 84.0444	Pyroglutamic acid^#^

10	1.12	268.1049	[M + H]^+^	C_10_H_13_N_5_O_4_	3.21	266.2041 205.1065 136.0608	Adenosine^#^

11	1.20	152.0572	[M + H]^+^	C_5_H_5_N_5_O	3.38	135.0291 110.0342	Guanine^#^

12	1.41	125.0233	[M − H]^−^	C_6_H_6_O_3_	−0.16	125.0233 81.0335	Maltol^#^

13	1.59	253.2150	[M − H]^−^	C_16_H_30_O_2_	−1.20	253.2150 128.0719 63.9018	Palmitelaidic acid^#^

14	1.91	125.0233	[M − H]^−^	C_6_H_6_O_3_	−0.16	81.0335 125.0233	Pyrogallol^#^

15	2.39	353.1425	[M − H]^−^	C_21_H_22_O_5_	4.19	197.1601 185.1158	Xanthohumol^#^

16	2.76	253.2162	[M − H]^−^	C_16_H_30_O_2_	−4.34	253.2162 199.9266 167.1201	Palmitoleic acid^#^

17	2.90	431.1213	[M − H]^−^	C_18_H_24_O_12_	4.18	431.1213 75.9928 59.0140	Asperulosidic acid^#^

18	3.02	211.0594	[M − H]^−^	C_10_H_12_O_5_	4.74	211.0594 127.7550 79.9591	Propyl gallate^#^

19	3.24	188.0710	[M + H]^+^	C_11_H_9_NO_2_	2.13	170.0590 146.0590 142.0642 118.0643	Trans‐3‐indoleacrylic acid^#^

20	3.26	367.1587	[M − H]^−^	C_19_H_28_O_5_S	−1.83	193.0489 191.0564 135.8035	Testosterone sulfate^#^

21	3.64	175.0608	[M − H]^−^	C_7_H_12_O_5_	−1.71	157.0493 131.0702 129.0546 115.0390 113.0593 85.0651	2‐Isopropylmalic acid^#^

22	3.66	353.0842	[M − H]^−^	C_16_H_18_O_9_	−2.51	228.9290 135.0436 124.7049	Chlorogenic acid^#^

23	4.62	121.0293	[M − H]^−^	C_7_H_6_O_2_	−1.65	121.0293 43.0175	4‐Hydroxybenzaldehyde^#^

24	4.65	172.0975	[M − H]^−^	C_8_H_15_NO_3_	1.74	172.0975 130.0862 58.0294	N‐Acetyl‐Leu^#^

25	4.66	167.1054	[M + H]^+^	C_10_H_14_O_2_	−1.22	167.1054 149.0962 121.1015	6‐Pentyl‐2H‐pyran‐2‐one^#^

26	4.82	1031.5469	[M − H]^−^	C_51_H_84_O_21_	4.60	987.5543 945.5416 783.4857 475.3800	Malonyl‐floralginsenoside Rd5/isomer^†^

27	6.10	861.4868	[M + HCOO]^−^	C_42_H_72_O_15_	1.74	391.2865 491.3762 663.4298 815.4826	Ginsenoside Re5^†^

28	6.18	137.0240	[M − H]^−^	C_7_H_6_O_3_	−2.92	136.8638 109.0303 93.3557	4‐Hydroxybenzoic acid^#^

29	6.42	831.4763	[M + HCOO]^−^	C_41_H_70_O_14_	1.92	653.4280 491.3747 391.2855	Vinaginsenoside R11^#^

30	6.45	961.5390	[M + HCOO]^−^	C_47_H_80_O_17_	2.44	637.4325 799.4848 475.3802	Ginsenoside Rd2^#^

31	6.50	1007.5455	[M + HCOO]^−^	C_48_H_82_O_19_	3.37	961.5394 799.4848 475.3812	Vinaginsenoside R8^#^

32	6.60	967.5057	—	—	—	227.7826	Unknown^†^

33	6.68	931.5284	[M − H]^−^	C_47_H_80_O_18_	1.29	783.4940 175.0569 161.0460	Notoginsenoside R1^#^

34	6.71	455.3485	[M − H]^−^	C_30_H_48_O_3_	−3.49	455.3485 409.2083 169.7267	Oleanolic acid isomer^†^

35	6.85	945.5438	[M − H]^−^	C_48_H_82_O_18_	1.06	945.5438 783.4916 637.4329 475.3782 161.0458	Ginsenoside Rd^∗^ ^,^ ^∗∗^

36	6.87	991.5498	[M + HCOO]^−^	C_48_H_82_O_18_	2.61	945.5400 783.4909 637.4334	Ginsenoside Re^∗^ ^,^ ^∗∗^

37	6.89	845.4914	[M + HCOO]^−^	C_42_H_72_O_14_	1.29	391.2869 161.0456 119.0355	Ginsenoside Rg1^∗^ ^,^ ^∗∗^

38	6.93	945.5432	[M − H]^−^	C_48_H_82_O_18_	0.42	783.4902 179.0561 161.0457	Notoginsenoside K^#^ ^,^ ^∗∗^

39	7.15	841.496	[M + HCOO]^−^	C_44_H_74_O_15_	1.90	781.4753 637.4320 475.3770 227.7986	Acetyl‐ginsenoside Rg1/isomer^†^

40	7.19	815.4817	[M + HCOO]^−^	C_41_H_70_O_13_	3.61	769.4751 637.4318 475.3801	Ginsenoside F3^#^

41	7.23	843.4767	[M + HCOO]^−^	C_42_H_74_O_14_	−1.54	635.4212 473.3631 227.7894 113.0246	(C_31_H_52_O_7_)‐glc‐rha^†^

42	7.29	847.5075	[M + HCOO]^−^	C_42_H_74_O_14_	2.32	635.4520 477.3971 421.7100 227.7943 101.0246	20(*S*)‐Ginsenoside Rf2^†^

43	7.41	885.4819	[M − H]^−^	C_45_H_74_O_17_	−2.38	841.4959 781.4800 637.4328 345.8713	Malonyl‐ginsenoside Rg1^†^

44	7.75	887.5022	[M + HCOO]^−^	C_44_H_74_O_15_	2.62	841.4965 781.4759 619.4245 161.0460	Notoginsenoside Rt^#^

45	7.91	1033.5603	[M + HCOO]^−^	C_50_H_84_O_19_	2.43	987.5546 945.5429 637.4332 619.4202	PPD‐3GLc‐ace^†^

46	8.13	1371.6800	[M − H]^−^	C_64_H_108_O_31_	−0.15	1371.6800 1329.6498 133.0149	Notoginsenoside D^†^

47	8.23	1239.6409	[M − H]^−^	C_59_H_100_O_27_	2.42	783.4907 621.4383 459.3863	Notoginsenoside R4^#^ ^,^ ^∗∗^

48	8.25	1341.6716	[M − H]^−^	C_63_H_106_O_30_	2.30	191.0564 323.0990 455.1399 1077.5759	Notoginsenoside‐Q^†^

49	8.31	1049.5529	—	—	—	1003.5499 961.5393 781.4751 799.4822	Unknown^†^

50	8.64	1209.6285	[M − H]^−^	C_58_H_98_O_26_	1.85	1049.5896 929.5474 915.5317	Ginsenoside Ra1^#^ ^,^ ^∗∗^

51	8.66	799.4858	[M − H]^−^	C_42_H_72_O_14_	2.46	799.4858 637.4332 475.3797	Pseudoginsenoside F11^∗^ ^,^ ^∗∗^

52	8.70	1239.6394	[M − H]^−^	C_59_H_100_O_27_	1.21	783.4907 621.4383 459.3863	Ginsenoside Ra3^#^ ^,^ ^∗∗^

53	8.70	1153.6001	[M + HCOO]^−^	C_54_H_92_O_23_	−0.87	1153.6001 945.5444 221.0662	Ginsenoside Rb1^∗^ ^,^ ^∗∗^

54	8.75	327.2162	[M − H]^−^	C_18_H_32_O_5_	−4.58	327.2162 177.165 137.0973	Corchorifatty acid F^#^

55	8.82	1193.6010	[M − H]^−^	C_57_H_94_O_26_	1.30	1149.6079 1089.5866 783.4913	Malonyl‐ginsenoside Rb1/isomer^†^

56	8.87	769.4756	[M − H]^−^	C_41_H_70_O_13_	1.56	769.4756 637.4327 161.0457	Ginsenoside F5^#^

57	8.91	1209.6294	[M − H]^−^	C_58_H_98_O_26_	1.65	1193.6319 1061.5896 1031.5791	Ginsenoside Ra2^#^ ^,^ ^∗∗^

58	8.97	955.4922	[M − H]^−^	C_48_H_76_O_19_	1.47	955.4922 793.4396 569.3855	Ginsenoside Ro^#^ ^,^ ^∗∗^

59	9.13	783.4911	[M − H]^−^	C_42_H_72_O_13_	1.41	161.0458 101.0246 85.0297	Ginsenoside C^#^

60	9.13	829.4976	[M + HCOO]^−^	C_42_H_72_O_13_	2.55	475.3794 783.3910	Ginsenoside Rg2/Rg3/isomer^#^

61	9.14	1077.5868	[M − H]^−^	C_53_H_90_O_22_	1.59	1077.5868 621.4377 459.3854	Ginsenoside Rb2^†^ ^,^ ^∗∗^

62	9.18	1077.5872	[M − H]^−^	C_53_H_90_O_22_	1.96	131.0351 191.0570 783.4876	Ginsenoside Rc^#^

63	9.25	1163.5888	[M − H]^−^	C_56_H_92_O_25_	2.05	227.7880 1059.5780 945.5573	Malonyl‐ginsenoside Rc/Rb2/isomer^†^

64	9.35	1149.6078	[M − H]^−^	C_56_H_94_O_24_	1.40	1089.5837 945.5454 783.4923	*O*‐Acetyl‐ginsenoside‐Rb1^†^

65	9.35	1195.6152	[M + HCOO]^−^	C_56_H_94_O_24_	3.72	1107.5956 1089.5803 783.4924 227.7989	Quinquenoside‐R1^†^

66	9.51	1251.6392	[M − H]^−^	C_60_H_100_O_27_	1.01	227.7877 765.4804 1077.5890	Ginsenoside Ra5/isomer^†^

67	9.58	1119.5980	[M − H]^−^	C_55_H_92_O_23_	2.02	783.4207 149.0471 459.3878 191.0564 173.0458	Ginsenoside Rs2/isomer^#^

68	9.69	637.4296	[M − H]^−^	C_36_H_62_O_9_	−3.92	637.4296 505.0657 225.8086	Ginsenoside Rh1^#^

69	9.70	683.4382	—	—	—	475.3797 227.7876 161.0466 113.0247 400.5006	Unknown^†^

70	9.73	683.4382	[M + HCOO]^−^	C_36_H_62_O_9_	0.92	475.4800 227.7869 161.0466 113.0249	Ginsenoside F1^†^

71	9.85	1165.5945	[M + HCOO]^−^	C_55_H_92_O_23_	−4.74	1119.5975 1077.5894 1059.5762	Ginsenoside Rs1^†^ ^,^ ^∗∗^

72	9.97	1119.5968	[M − H]^−^	C_55_H_92_O_23_	0.92	1077.5875 1059.5778 945.5394 783.4893	Ginsenoside Rs2/isomer^#^

73	10.10	1191.6188	[M + HCOO]^−^	C_57_H_94_O_23_	1.68	1107.5984 1071.5763 945.5473	Ginsenoside Ra7/Ra8/Ra9^†^

74	10.10	1237.608	—	—	—	1089.5867 1149.6062 945.5380	Unknown^†^

75	10.43	1191.6188	[M + HCOO]^−^	C_57_H_94_O_23_	1.68	1077.5892 945.5400 161.0462	Ginsenoside Ra7/Ra8/Ra9^†^

76	10.45	987.5548	[M − H]^−^	C_50_H_84_O_19_	1.42	927.5346 621.4379 783.49	Malonyl floralginsenoside Re1^†^

77	10.54	1207.6122	—	—	—	1119.5889 1077.583 1059.5863 998.6083	Unknown^†^

78	10.54	915.5324	[M − H]^−^	C_47_H_80_O_17_	0.14	915.5324 607.4024 427.7909	Notoginsenoside Fe^#^

79	10.56	1073.5554	—	—	—	945.5468 987.5507	Unknown^†^

80	11.03	1059.5747	[M + HCOO]^−^	C_52_H_86_O_19_	0.16	945.5454 783.4847 227.7873	Quinquenoside I^†^

81	11.35	1003.5508	—	—	—	957.5435 915.5354 783.4912 621.4383 227.7875	Unknown^†^

82	11.38	829.4970	[M + HCOO]^−^	C_42_H_72_O_13_	1.82	783.4912 621.437 161.046	Ginsenoside Rg2/Rg3/isomer^#^

83	11.56	665.427	[M + HCOO]^−^	C_36_H_60_O_8_	−0.03	517.6613 275.1061 109.609 81.1308	Ginsenoside Rh4/isomer^†^

84	11.71	1029.5656	—	—	—	945.543 909.5294 765.4851 621.4391	Unknown^†^

85	11.71	1075.5679	[M − H]‐	C_53_H_88_O_22_	−1.45	1029.5651 945.5450 945.545 987.5508	459‐glc‐glc‐glc‐2acetyl^†^

86	11.79	665.4270	[M + HCOO]^−^	C_36_H_60_O_8_	−0.03	517.6613 275.1061 109.6090 81.1308	Ginsenoside Rh4^∗^

87	11.82	318.2987	[M + H]^+^	C_18_H_39_NO_3_	−4.89	318.2987 300.2862 256.2622	2‐Amino‐1,3,4‐octadecanetriol^#^

88	12.16	829.4969	[M + HCOO]^−^	C_42_H_72_O_13_	1.69	783.4912 621.4369 161.0460	Ginsenoside Rg2/Rg3/isomer^#^

89	12.17	783.4910	[M − H]^−^	C_42_H_72_O_13_	2.66	783.4910 621.4384 459.3847	Ginsenoside F2^#^

90	12.63	311.2231	[M − H]^−^	C_18_H_32_O_4_	1.04	311.2231 293.2130 183.0130	9‐HPODE^#^

91	12.77	265.1484	[M − H]^−^	C_12_H_26_O_4_S	1.86	96.9603 265.1484	Dodecyl sulfate^†^

92	12.83	827.4812	[M + HCOO]^−^	C_42_H_70_O_13_	1.64	496.7871 311.7790 221.0673 179.0564 119.0354 101.0246	Ginsenoside Rg8/Rg9^†^

93	12.87	799.4858	[M − H]^−^	C_42_H_72_O_14_	1.10	765.4808 675.0261 603.4275	Ginsenoside Rf^#^

94	13.24	825.5016	[M − H]^−^	C_44_H_74_O_14_	1.24	765.4782 621.4362 459.3858 161.0460	20‐Ginsenoside‐Rs3^†^

95	13.24	871.5093	[M + HCOO]^−^	C_44_H_74_O_14_	3.73	783.4924 621.4388 101.0245	Ginsenoside Rs3^†^

96	13.48	285.2076	[M − H]^−^	C_16_H_30_O_4_	1.65	285.2076 267.1973 241.2184 223.2073	Hexadecanedioic acid^#^

97	14.00	765.4807	[M − H]^−^	C_42_H_70_O_12_	1.63	765.4807 675.0261 603.4275	Ginsenoside Rg5 isomer^†^

98	14.12	621.4365	[M − H]^−^	C_36_H_62_O_8_	−1.11	621.4365 147.4954 125.4886	20‐Ginsenoside Rh2^#^

99	14.14	667.4427	[M + HCOO]^−^	C_36_H_62_O_8_	0.05	621.4376 459.3811 161.0457	Ginsenoside Rh2^∗^

100	14.34	765.4804	[M − H]^−^	C_42_H_70_O_12_	1.24	765.4804 675.0261 603.4275	Ginsenoside Rg5^∗^

101	14.66	295.2284	[M − H]^−^	C_18_H_32_O_3_	1.83	277.2178 113.0974 59.0140	13‐HODE^#^

102	14.93	665.427	[M + HCOO]^−^	C_36_H_60_O_8_	−0.03	517.6613 275.1061 109.6090 81.1308	Ginsenoside Rh4/isomer^†^

103	15.59	853.4867	—	—	—	807.4911 747.4745 227.7876	Unknown^†^

104	15.69	807.4913	[M − H]^−^	C_44_H_72_O_13_	1.60	603.4277 227.7781 161.0457 113.0246 101.0246	Ginsenoside Rs5/Rs4^†^

105	15.95	1093.5798	[M − H]^−^	C_53_H_90_O_23_	−0.19	1077.5865 945.5426 783.4880	Ginsenoside Rb3^#^

106	16.77	813.5057	—	—	—	729.2033 283.0531 227.7979 161.0465	Unknown^†^

107	17.30	271.2282	[M − H]^−^	C_16_H_32_O_3_	2.25	271.2282 253.2189 225.2227	16‐Hydroxyhexadecanoic acid^#^

108	17.58	301.2177	[M − H]^−^	C_20_H_30_O_2_	1.33	301.2177 257.2261 203.1809	Eicosapentaenoic acid^#^

109	17.74	455.3535	[M − H]^−^	C_30_H_48_O_3_	0.97	455.3535 409.2083 169.7267	Oleanolic acid^#^ ^,^ ^∗∗^

110	17.80	277.2175	[M − H]^−^	C_18_H_30_O_2_	0.72	277.2175 233.2261 59.0132	Pinolenic acid^#^

111	18.70	282.2794	[M + H]^+^	C_18_H_35_NO	0.92	282.2794 265.2905 247.2406	Oleamide^#^

112	20.26	281.2490	[M − H]^−^	C_18_H_34_O_2_	1.42	281.2490 237.1366 83.5180	Oleic acid^#^

113	21.66	283.2646	[M − H]^−^	C_18_H_36_O_2_	1.24	283.2646 265.0729 97.4590	Stearic acid^#^

^∗^Compound is a standard.

^#^The compound has been cross‐referenced with the HMDB and PubChem databases.

^†^The ccompound was identified by GNPS.

^∗∗^The compound is a blood‐absorbed ingredient.

### 3.5. Analysis of the Blood‐Absorbed Components of GDP

The TICs of blank plasma and postadministration plasma samples at different time points were simultaneously analyzed according to the conditions described in Section 2.4. By combining previously characterized chemical constituents of GDP with chromatographic *t*_*R*_ and MS/MS fragmentation patterns, 13 prototype components were tentatively identified as absorbed components. To more clearly illustrate the differences between GDP and plasma samples, the TICs were overlaid (Figure 5(a)). The corresponding chromatographic peaks of these specific components in GDP and plasma after administration are shown in Figure 5(b). To visualize the abundance patterns of these 13 absorbed components across individual rats at different time windows (0‐1 h and 2–4 h, *n* = 5 rats), a heatmap of peak areas was generated (Figure S12).

**FIGURE 5:**
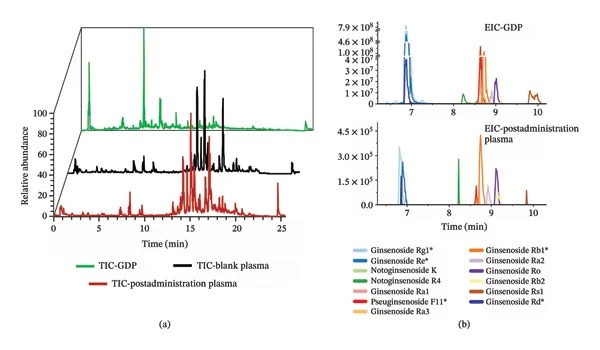
(a) TICs of GDP, blank plasma, plasma after administration, and blood‐absorbed ingredients in negative ionization mode. (b) EICs of GDP and postadministration plasma samples.

### 3.6. Network Pharmacology Results

#### 3.6.1. PPI Network of Overlapping Targets Between Bioactive Compounds and CVD‐Related Genes in GDP

Disease target databases identified 2573 potential therapeutic targets for CVD, while drug target analysis revealed 174 drug component targets associated with GDP’s bioactive constituents. Venn analysis identified 100 overlapping targets, which were considered candidate targets potentially involved in the therapeutic effects of GDP against CVD (Figure 6(a)). A PPI network was constructed from these overlapping targets (Figure S11) and analyzed in Cytoscape. Nodes were ranked according to network centrality parameters, including degree, betweenness, and closeness. The top 18 nodes with the highest connectivity and centrality, including Prostaglandin endoperoxide synthase 2 (PTGS2), peroxisome proliferator–activated receptor gamma (PPARG), Mitogen‐activated protein kinase 3 (MAPK3), and Signal transducer and activator of transcription 3 (STAT3), were identified as core targets and highlighted in Figure 6(b). These topologically important nodes may represent key targets mediating the pharmacological effects of GDP on CVD. Integrating these findings, we constructed a GDP–bioactive compounds–key targets–pathways–CVD interaction network to provide a systems‐level view of the predicted interactions (Figure 6(c)).

FIGURE 6(a) Venn diagram of intersecting targets between GDP and CVD. (b) Core target analysis of GDP. (c) GDP–bioactive compounds–key targets–pathways–disease network diagram for the treatment of CVD. (d) GO analysis, top 10 GO terms for BP, CC, and MF categories. (e) KEGG pathway enrichment analysis.

**Figure:**
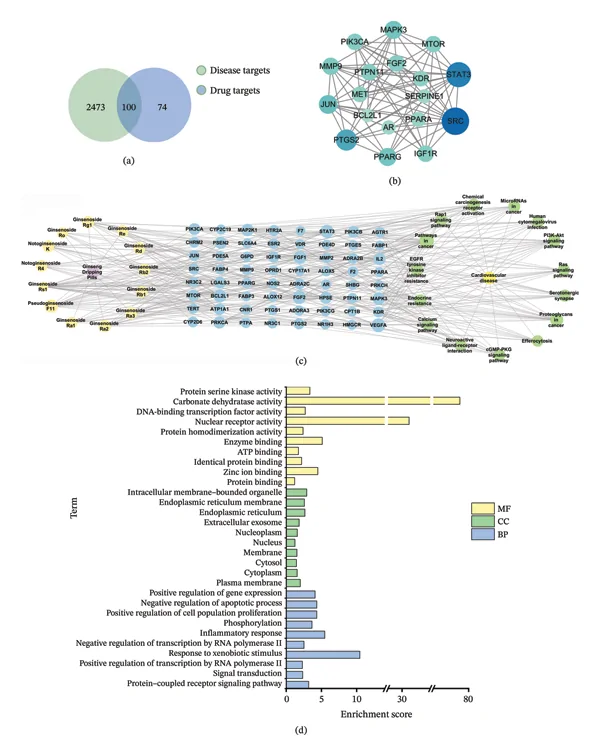

**Figure:**
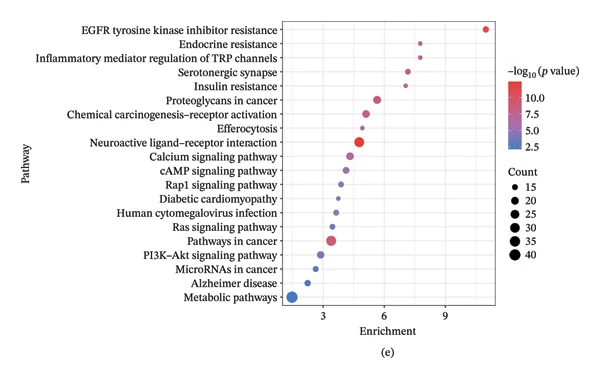

#### 3.6.2. Comparative Analysis of GDP and CVD Based on Network Construction

Based on topological parameters, including degree, closeness centrality, and betweenness centrality, five key active ingredients in GDP were identified (Table 2), which may play significant roles in its potential therapeutic effect on CVD. Based on these findings, we constructed a GDP–bioactive compounds–targets–CVD interaction network (Figure 6(c)) comprising 100 nodes and 406 edges. The analysis revealed that key compounds, including pseudoginsenoside F11, ginsenoside Rg1, ginsenoside Ra3, notoginsenoside R4, and ginsenoside Ro, may be associated with the regulation of the phosphatidylinositol 3‐kinase (PI3K)–Akt signaling pathway through predicted core targets: PTGS2, MAPK3, STAT3, Matrix metalloproteinase 9 (MMP9), and Plasminogen activator inhibitor‐1 (SERPINE1). Furthermore, the network indicated that PI3K‐Akt, renin–angiotensin system (RAS), cyclic adenosine monophosphate (cAMP), Ras‐related protein 1 (Rap1), and calcium signaling pathways are potentially involved in the pharmacological actions.

**TABLE 2:** Topological information of the key active ingredients in GDP.

Serial number	Active ingredient	Degree	Closeness centrality	Betweeness centrality
1	Pseudoginsenoside F11	31	0.005154639	2911.064909
2	Ginsenoside Rg1	19	0.004587156	700.2555047
3	Ginsenoside Ra3	14	0.004310345	192.3427845
4	Notoginsenoside R4	14	0.004310345	192.3427845
5	Ginsenoside Ro	10	0.004166667	208.0741407

#### 3.6.3. GO and KEGG Enrichment Pathway Analyses of Overlapping Genes

Figures 6(d and e) present the top 10 significantly enriched GO terms (*p* < 0.05) and top 20 enriched KEGG pathways (*p* < 0.05). In Figure 6(d), GO analysis revealed that enriched BP terms were related to apoptosis regulation, inflammatory response, xenobiotic response, and signal transduction, suggesting GDP may be involved in the regulation of cell survival and inflammation, consistent with its cardioprotective effect. The CC terms were primarily localized to membrane‐related structures, including plasma membrane, endoplasmic reticulum, extracellular exosome, and nucleoplasm, highlighting the involvement of receptor‐mediated signaling and intercellular communication in GDP’s pharmacological activity. MF terms involved binding and enzymatic activities, including kinase activity, DNA‐binding transcription factors, and nuclear receptor activity, suggesting potential involvement in transcriptional regulation, receptor activity, and kinase‐mediated signaling. The plasma membrane localization and receptor binding functions further substantiate CVD therapeutic targeting through key pathways such as PI3K‐Akt and cAMP signaling.

KEGG pathway analysis identified 116 significantly enriched pathways (*p* < 0.05), with the top 20 illustrated in Figure 6(e). Following exclusion of cancer‐related pathways, the findings suggest that GDP may influence CVD progression via multiple signaling pathways, including PI3K‐Akt, RAS, cAMP, Rap1, and calcium signaling. These are known to be involved in key cardiovascular processes including cellular repair/regeneration, vasomotor tone modulation, and blood pressure homeostasis.

#### 3.6.4. Molecular Docking Analysis

From the 18 core targets identified by PPI network analysis, seven proteins (JUN, MAPK3, MMP9, PPARG, PTGS2, SRC, and STAT3) were selected for molecular docking based on their topological importance and well‐documented roles in CVD. Molecular docking was then performed between these targets and the main bioactive components of GDP. As shown in Figure 7(a), a total of 91 docking results were obtained. Ginsenoside Ra3–PTGS2 and ginsenoside Ro–MAPK3 complexes demonstrated the lowest binding energies, indicating potentially strong affinities (visualized in Figure 7(b)). In contrast, c‐Jun (JUN) exhibited weaker binding interactions. According to widely adopted criteria in network pharmacology studies, binding energies < −6.0 kcal/mol indicate favorable binding activity, with more negative values corresponding to greater binding stability. Almost all compound–target pairs satisfied this criterion.

**FIGURE 7:**
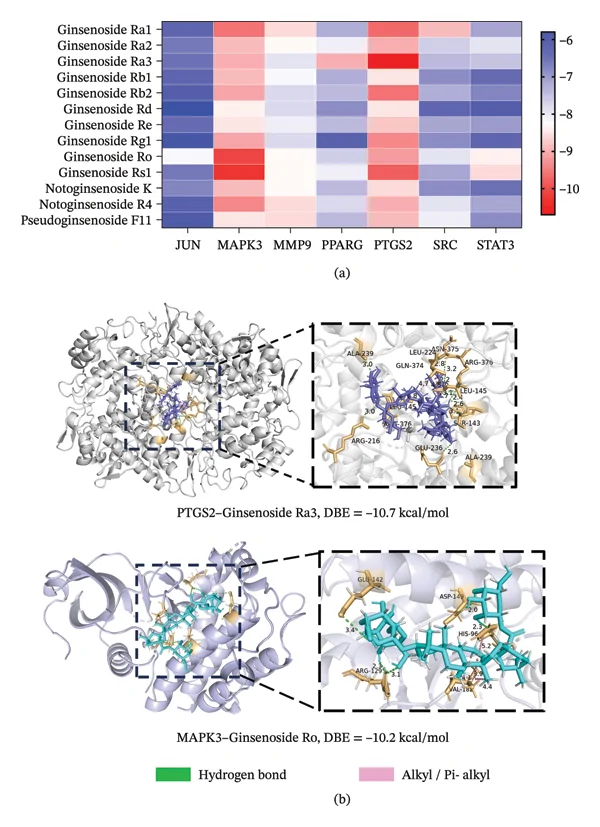
Molecular docking results of GDP. (a) Heatmap of molecular docking binding energies. (b) Visualization of molecular docking results for key ginsenoside bioactive compounds with receptor protein complexes.

To further validate the reliability of the docking results, redocking analysis was performed for representative complexes. The RMSD values for MAPK3–ginsenoside Ro, MAPK3–ginsenoside Rs1, and PTGS2–ginsenoside Ra3 were 0.176 Å, 0.911 Å, and 0.358 Å, respectively. All RMSD values were well below 2.0 Å, indicating good reproducibility and stability of the docking poses. Overall, these results suggest that bioactive constituents of GDP may interact with multiple cardiovascular‐related targets, particularly those involved in inflammatory regulation, although further experimental validation is required.

## 4. Discussion and Conclusions

Ginseng, the primary bioactive source in GDP, has been traditionally employed in TCM for CVD management. Modern pharmacological studies have demonstrated its wide range of therapeutic effects, including inhibition of myocardial fibrosis, prevention of myocardial ischemia/reperfusion injury, antiarrhythmic effects, and antiatherosclerotic effects. It is widely used in treating heart failure, arrhythmia, and unstable angina pectoris [33–35]. GDP is derived from *Panax ginseng*, and distinguishing its chemical features from other *Panax* species is relevant for quality evaluation. Among the 11 *Panax* species, *Panax ginseng* and *Panax quinquefolius* are the most pharmacologically significant, cultivated mainly in China, Korea, and North America. Although they share similar total ginsenoside contents and comparable levels of PPD‐type ginsenosides, they differ in chemical composition. Ginsenoside Rf is characteristic of *Panax ginseng*, whereas F11 is unique to *Panax quinquefolius*. In addition, variations in ginsenoside profiles and the Rg1:Rb1 ratio provide reliable parameters for species differentiation [36–38]. These chemotaxonomic markers can aid in verifying the species origin of ginseng preparations.

In the present study, UHPLC‐Q‐Orbitrap‐MS analysis identified 113 chemical components in GDP, with ginsenosides as the predominant constituents. Blood‐absorbed component analysis is a widely adopted approach for identifying active TCM components [39]. This study detected 13 prototype compounds in rat plasma, suggesting favorable absorption and metabolic properties in vivo. Among these, ginsenosides Rg1, Re, Rb1, and Rd are well‐known plasma‐exposed ginsenosides after oral administration of ginseng preparations [40], and ginsenosides Ra3, Rb1, Rd, and Rg1 have also been detected in human plasma following Sanqi extract intake [41]. Pseudoginsenoside F11 has been shown to undergo metabolism in rats and protect against thromboembolic stroke injury by reducing thromboinflammation [42], suggesting its cardiovascular relevance. Ginsenoside Ro possesses anti‐inflammatory and antiplatelet properties and has been observed in plasma after oral ginseng administration [43, 44]. In contrast, notoginsenoside K, notoginsenoside R4, and ginsenoside Ra1/Ra2 were less frequently reported as plasma‐absorbed components, possibly due to differences in formulation or analytical sensitivity. Nevertheless, notoginsenoside R4 has been predicted to interact with STAT3 and AKT1 in docking analyses [45], and notoginsenoside K exhibits hemolytic and adjuvant activities [46], suggesting potential biological relevance.

Network pharmacology analysis identified 18 core CVD‐related targets, including PTGS2, PPARG, MAPK3, STAT3, MMP9, and SRC, which are critically involved in the pathogenesis and progression of CVD. For instance, PTGS2 plays a pivotal role in inflammation and thrombosis [47], while MAPK3 regulates cell proliferation, differentiation, and apoptosis through multiple signaling cascades [48]. GO and KEGG pathway enrichment analyses suggested that GDP may exert its therapeutic effects primarily through the PI3K‐Akt, RAS, cAMP, Rap1, and calcium signaling pathways, which regulate cell proliferation, apoptosis, inflammation, and neovascularization. For example, the PI3K‐Akt pathway is known to be crucial for cell survival and growth, with its dysregulation having been linked to AS and MI [49]. These pathway findings agree with published reports: ginsenoside Rd attenuates myocardial ischemia/reperfusion injury via PI3K/Akt activation [50], and a systems pharmacology study on ginseng against heart failure also highlighted PI3K‐Akt and AMPK pathways as primary mediators [51]. Furthermore, the core targets PTGS2, MAPK3, and STAT3 have been consistently identified in network pharmacology studies of ginsenosides. The involvement of MAPK and NF‐κB cascades in ginsenoside‐mediated cardiovascular protection has been comprehensively reviewed [52]. In addition, a combined network pharmacology and proteomics study demonstrated that ginsenoside Re exerts its effects on myocardial ischemia by regulating MAPK, AKT, and STAT3 signaling pathways [53].

The molecular docking results indicated that nearly all compound–target pairs exhibited binding energies below −6.0 kcal/mol. Notably, PTGS2 exhibited the lowest binding energy with each active ingredient, suggesting its potential role in CVD. To further validate the reliability of the docking results, redocking analysis was performed for representative ligand–target complexes. The RMSD values for MAPK3–ginsenoside Ro, MAPK3–ginsenoside Rs1, and PTGS2–ginsenoside Ra3 were 0.176 Å, 0.911 Å, and 0.358 Å, respectively, all well below 2.0 Å. These results indicate good reproducibility and stability of the predicted binding poses. Overall, these findings suggest that bioactive constituents of GDP may interact with multiple cardiovascular‐related targets.

Although this study systematically characterizes the chemical composition of GDP and explores its potential mechanisms using multiple modern analytical approaches, it still has several limitations. First, the predictions derived from network pharmacology and molecular docking require further validation through in vitro and in vivo experiments to confirm the interactions between bioactive compounds and their targets. Second, although the rat study provided valuable information on absorbed constituents, the pharmacological effects of GDP and the roles of these absorbed components were not further validated in CVD models. Therefore, the findings should be considered tentative. Third, the sample size for plasma analysis was relatively small (*n* = 5). This study was exploratory, aiming to screen for absorbed components rather than to make statistical comparisons. The sample size follows common practice for early‐stage profiling. Nevertheless, the limited sample size may reduce generalizability, so future validation in larger cohorts is needed. In addition, future investigation of other potential active constituents and their mechanisms of action will contribute to a more comprehensive understanding of the pharmacological effects of GDP.

In summary, this study systematically explored the potential mechanisms underlying the cardiovascular protective effects of GDP using modern analytical techniques. It further provided computational evidence suggesting that GDP may be involved in the regulation of multiple signaling pathways. These findings not only provide a preliminary theoretical basis for further clinical application of GDP, but also offer valuable insights for the future modernization and development of TCM.

**Nomenclature**
AS: Atherosclerosis
ASCVD: Atherosclerotic cardiovascular disease
BPs: Biological processes
cAMP: Cyclic adenosine monophosphate
CCs: Cellular components
CH_3_CN: Acetonitrile
CVD: Cardiovascular disease
DAVID: The Database for Annotation, Visualization, and Integrated Discovery
DBE: Docking binding energy
FA: Formic acid
GDPs: Ginseng dripping pills
GNPS: The Global Natural Products Social Platform
GO: Gene Ontology
HESI: Heated electrospray ionization
HMDB: The Human Metabolome Database
HOAc: Acetic acid
KEGG: Kyoto Encyclopedia of Genes and Genomes
LC‐MS: Liquid chromatography–mass spectrometry
MAPK3: Mitogen‐activated protein kinase 3
MeOH: Methanol
MFs: Molecular functions
MI: Myocardial infarction
N_2_: Nitrogen
NCE: Normalized collision energy
OA‐type: Oleanolic acid–type
OMIM: Online Mendelian Inheritance in Man database
PDB: Protein Data Bank database
PEG: Polyethylene glycol
PI3K‐Akt: Phosphatidylinositol 3‐kinase
PPARG: Peroxisome proliferator–activated receptor gamma
PPD‐type: Protopanaxadiol‐type
PPI: Protein–protein interaction
PPT‐type: Protopanaxatriol‐type
PTGS2: Prostaglandin endoperoxide synthase 2
RAS: Renin–angiotensin system
SPF: Specific pathogen–free
STAT3: Signal transducer and activator of transcription 3
TCM: Traditional Chinese medicine
TIC: Total ion chromatogram
*t*_*R*_: Retention times
TTD: Therapeutic Target Database
UHPLC‐Q‐Orbitrap MS: Ultra‐high performance liquid chromatography–quadrupole‐Orbitrap mass spectrometry

## Author Contributions

Minglin Xu: writing–original draft and data curation. Junyi Mao: software and data curation. Xiangnan Wang: visualization. Jinping Jiang: data curation. Simon Sani Ocholi: writing–review and editing. Chaoyang Wang: visualization and investigation. Yue Wang: supervision and methodology. Honghai Yu: supervision and funding acquisition. Lifeng Han: writing–review and editing and supervision, Funding acquisition.

## Funding

This study was supported by the Tianjin Committee of Science and Technology of China (Grant no. 24ZYJDSS00310) and the National Natural Science Foundation of China (Grant no. 82411540242).

## Conflicts of Interest

The authors declare no conflicts of interest.

## Supporting Information

Additional supporting information can be found online in the Supporting Information section.

## Supporting information


**Supporting Information** Figure S1: Optimization of chromatographic column conditions: spectrum of condition optimization and number of peaks extracted. Figure S2: Optimization of aqueous phase species for BEH C_18_ columns: spectrum of condition optimization and number of peaks extracted. Figure S3: Optimization of acetic acid concentration on BEH C_18_ column in chromatographic separation: spectrum of condition optimization and number of peaks extracted. Figure S4: Optimization of different column temperatures for BEH C_18_ column in chromatographic separation: spectrum of condition optimization and number of peaks extracted. Figure S5: Optimization of different flow rates on BEH C_18_ columns for chromatographic separations. Figure S6: TIC of GDP obtained using the optimized UHPLC‐Q‐Orbitrap MS method with a total run time of 25.5 min. >Figure S7–S10: GNPS molecular network diagram of GDP. Figure S11: PPI network of blood‐absorbed ingredients for CVD therapy. Figure S12: Heatmap of log_10_‐transformed peak areas of blood‐absorbed components in rat plasma.

## Data Availability

All data included in this study are available upon request by contacting the corresponding author.
