# Supplementary material for: Exploration of Active Compounds and Potential Mechanisms of Ginseng Dripping Pills: An Integrated Study Utilizing UHPLC‐Q‐Orbitrap MS Analysis and Network Pharmacology
Source: Int J Anal Chem. 2026 Sep 28;2026:6693812. doi: 10.1155/ianc/6693812 (PMC13618928; doi:10.1155/ianc/6693812)
Supplement: Supplementary file 1 [file IANC-2026-6693812-s001.docx]

Figure S1 Optimization of chromatographic column conditions: Spectrum of condition optimization and number of peaks extracted

Figure S2 Optimization of aqueous phase species for BEH C_18_ columns: Spectrum of condition optimization and number of peaks extracted

Figure S3 Optimization of acetic acid concentration on BEH C_18_ column in chromatographic separation: Spectrum of condition optimization and number of peaks extracted

Figure S4 Optimization of different column temperatures for BEH C_18_ column in chromatographic separation: Spectrum of condition optimization and number of peaks extracted

Figure S5 Optimization of different flow rates on BEH C_18_ columns for chromatographic separations

Figure S6 TIC of GDP obtained using the optimized UHPLC-Q-Orbitrap-MS method with a total run time of 25.5 min

Figure S7 GNPS molecular network diagram of GDP

Figure S8 GNPS molecular network diagram of GDP

Figure S9 GNPS molecular network diagram of GDP

Figure S10 GNPS molecular network diagram of GDP

Figure S11 PPI network of blood-absorbed ingredients for CVD therapy

Figure S12 Heatmap of log_10_-transformed peak areas of blood-absorbed components in rat plasma
